# Detection of inguinal lymph nodes is promising for the diagnosis of periprosthetic joint infection

**DOI:** 10.3389/fcimb.2023.1129072

**Published:** 2023-04-28

**Authors:** Leilei Qin, Chen Zhao, Hai Wang, Jianye Yang, Li Chen, Xudong Su, Li Wei, Tao Zhang, Jia Li, Changchun Jian, Ning Hu, Wei Huang

**Affiliations:** ^1^Department of Orthopaedics, The First Affiliated Hospital of Chongqing Medical University, Chongqing, China; ^2^Orthopedic Laboratory, Chongqing Medical University, Chongqing, China; ^3^Department of Orthopaedics, Fuling Central Hospital Affiliated of Chongqing University, Chongqing, China; ^4^Department of Radiology, The First Affiliated Hospital of Chongqing Medical University, Chongqing, China; ^5^Department of Orthopedics, Affiliated Hospital of North Sichuan Medical College, Sichuan, China

**Keywords:** prosthetic joint infection, lymph nodes, lymphadenopathy, ultrasound, diagnosis

## Abstract

**Background:**

Localized inguinal lymphadenopathy often represents lower extremity pathogen infection, while normalized lymphadenopathy is associated with infection regression. We hypothesized that inguinal lymph nodes (LNs) were enlarged in Periprosthetic Joint Infection (PJI) patients and that normalized inguinal LNs would be a promising way to determine the timing of reimplantation.

**Methods:**

We prospectively enrolled 176 patients undergoing primary and revision hip or knee arthroplasty. All patients underwent ultrasound examination of inguinal LNs preoperatively. The diagnostic value of inguinal LNs in PJI was evaluated by the receiver operating characteristic (ROC) curve.

**Results:**

The median level of inguinal LNs was 26mm in the revision for PJI group compared with 12 mm in the aseptic revision group (p< 0.0001). The size of the inguinal LNs well distinguishes PJI from aseptic failure (AUC= 0.978) compare with ESR (AUC= 0.707) and CRP (AUC= 0.760). A size of 19mm was determined as the optimal threshold value of the inguinal LNs for the diagnosis of PJI, with a sensitivity of 92% and specificity of 96%.

**Conclusion:**

Ultrasonic analysis of inguinal LNs is a valuable piece of evidence for the diagnosis of PJI and evaluation of persistent infection.

## Introduction

Periprosthetic joint infection (PJI) after total joint arthroplasty is still one of the catastrophic complications that clinicians must overcome (Kurtz et al., 2012; Parvizi et al., 2018). Accurate diagnosis of PJI is related to the surgical method of prosthesis revision and the optimal timing of two-stage exchange arthroplasty. However, timely and accurate diagnosis of PJI remains a challenge, as PJI are often caused by low-virulence pathogens and are associated with mature prosthetic biofilms (Qin et al., 2020). Currently, the diagnosis of PJI relies on molecular biomarkers derived from serum and synovial fluid in addition to conventional etiological tests (Cheok et al., 2022; Goud et al., 2022). Meanwhile, with the development of molecular biology research, the application of some new technologies, such as fluorescence *in situ* hybridization and next-generation sequencing technology, has significantly reduced the difficulty of PJI diagnosis (Lippmann et al., 2019; Kildow et al., 2021). The use of novel biomarkers and specific testing techniques remains controversial, considering the availability of tests in different healthcare facilities and the cost-effectiveness of these tests. Therefore, it is a common goal of clinicians to find a noninvasive test with a high recognition effect for PJI.

The lymphatic system is part of the body’s immune system. Of these, lymph nodes are accessory structures to the entire lymphatic system and play a crucial role in the body’s ability to fight infection, acting as filters for foreign bodies such as cancer cells and infections (Zhang et al., 2021). The cells in the lymph nodes are lymphocytes, which produce antibodies (protein particles that bind to foreign substances, including infectious particles), and macrophages that digest debris, which act as the body’s “cleaning” cells (Girard and Springer, 1995). Swollen lymph nodes usually occur as a result of infection from bacteria or viruses (Shimono et al., 2017). Among them, inguinal lymph nodes serve as guardians of lower limb immunity, and their pathological enlargement may indicate an infection in the lower body (Bui and Bordoni, 2022). Inguinal lymph nodes in asymptomatic patients have a mean short axis of 5.4 mm, usually no more than 10mm, and a short axis diameter greater than 15 mm is considered abnormal (Bontumasi et al., 2014). Notably, lymph nodes rapidly increase in size in response to infection in adjacent tissues and organs within 2 to 3 days and return to normal size within 2 to 4 weeks after the infection is fully controlled (Indelicato et al., 2006; Shimono et al., 2017).

We hypothesized that the severity of inguinal lymphadenopathy is different from pathogen-induced PJI and sterile inflammatory induced prosthetic aseptic failure, and such difference is beneficial for differentiating between different disease types. Therefore, our purpose was to explore whether ultrasound assessment of inguinal lymph node size can be used for diagnosing PJI. Additionally, we also investigated the performance of inguinal lymph nodes in patients undergoing reimplantation, which is of interest in determining whether infection persists before reimplantation.

## Materials and methods

### Patient cohort and characteristics

**Figure 1:**
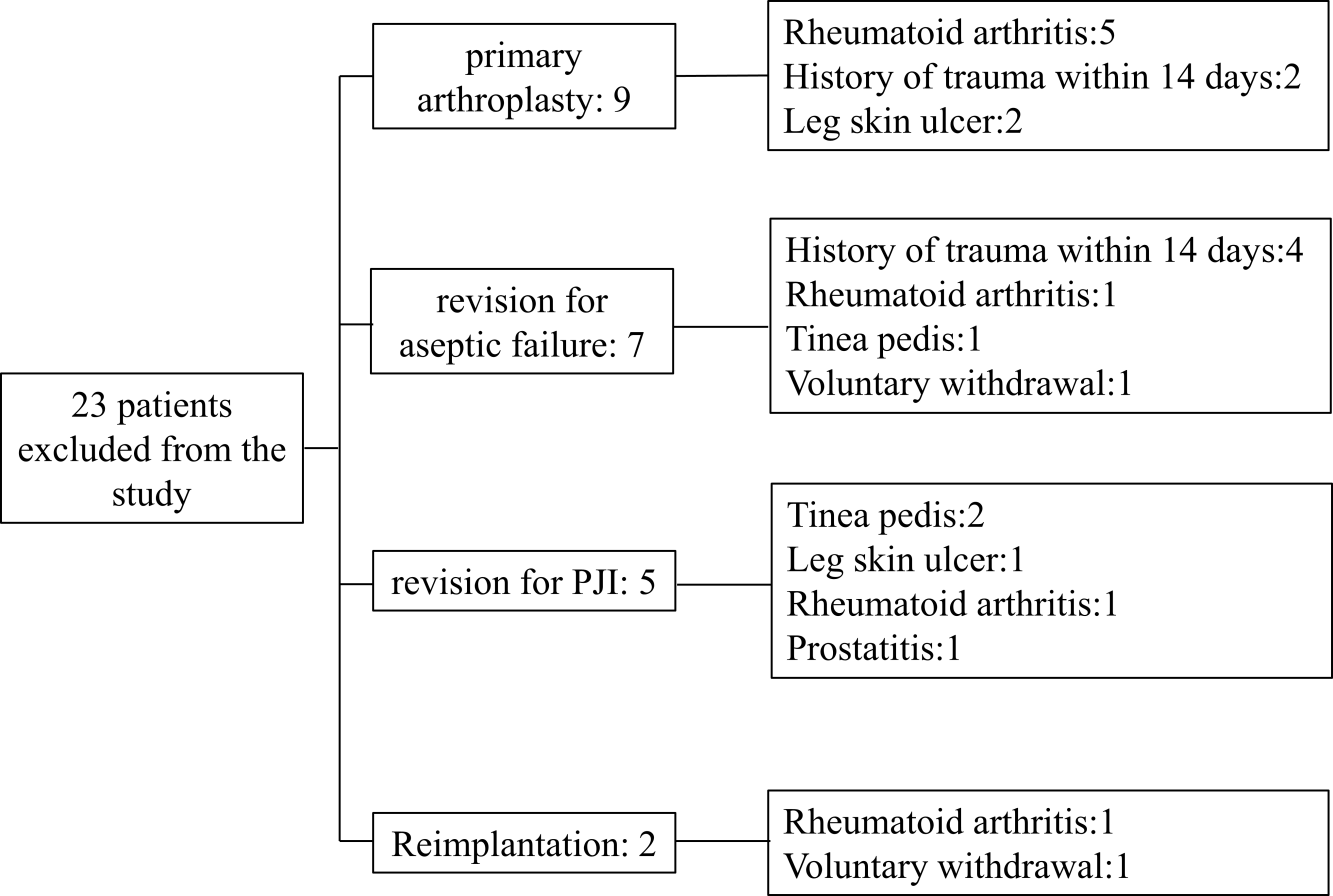
The number and reasons of excluded patients, by group.

From January 2020 to June 2022, we prospectively included patients who underwent primary total hip/knee arthroplasty (TJA) or hip/knee revision procedures. Patients with any type of skin ulcers, hematoma, sexually transmitted diseases (STDs), a recent history of trauma or dislocation (within 2 weeks), extra-articular infections of the lower extremities, co-existing immune system diseases such as rheumatoid arthritis, tumors (lymphoma, skin cancer of the lower extremities, prostate cancer, bladder cancer, and gynecological tumors), and those receiving immunotherapy were excluded. Patients included in this study were divided into group A: patients receiving primary arthroplasty; Group B: patients who underwent revision arthroplasty due to aseptic failure; Group C: patients undergoing the first stage of a 2-stage exchange revision protocol for the treatment of PJI; Group D: patients treated with PJI undergoing reimplantation protocol. For the reimplantation group, we first confirmed that every patient had no pain or discomfort regarding to clinical signs. Then we conducted MSIS standards and synovial fluid culture to exclude the possibility of infection. During the operation, two surgeons with more than 20 years of experience took different tissues for microbial culture. The cohort was included in the study after a series of methods failed to detect signs of infection. A total of 23 patients were excluded for reasons including rheumatoid arthritis (8 patients), a history of trauma within 14 days (6 patients), leg ulcers (3 patients), tinea pedis (3 patients), prostatitis (1 patient), and voluntary withdrawal from the cohort (2 patients). The number of exclusions and the specific reasons are recorded in **Figure 1**.

Aseptic revision refers to the failure of primary arthroplasty due to loosening, wear, instability, and dislocation of the prosthesis, excluding infection (Qin et al., 2020; Qin et al., 2022). The procedure is based on history, physical examination, imaging, serum inflammatory markers, intraarticular synovial fluid sampling, and intraoperative visual evaluation of the prosthesis with pain during movement and presence of the bone-cement interface around the prosthesis. Indications of implant loosening including 2mm opacity, osteolysis, implant displacement, and heterotopic ossification (Chalmers et al., 2017; Anil et al., 2022). PJI is defined by the Musculoskeletal Infection Society (MSIS) criteria (Parvizi and Gehrke, 2014). We recorded gender, age, the involved joint and BMI of the patients. At least 3 tissue samples were collected for microbiological culture and extended culture for 14 days during revision arthroplasty.

### Procedure for detecting inguinal lymph nodes by ultrasound

**Figure 2:**
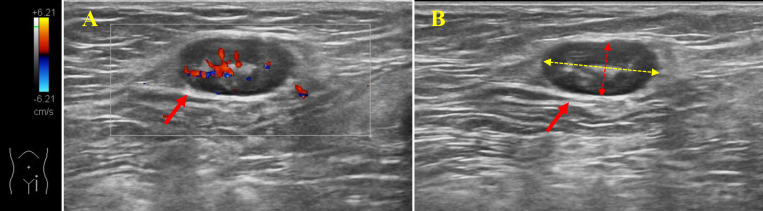
Ultrasonography of inguinal lymph nodes. **(A)**: Color Doppler shows enlarged blood supply in lymph nodes in the inguinal region. **(B)**: Measurements of the long and short axes of the lymph nodes. Solid red arrows: lymph nodes; Yellow dotted arrow: long axis of lymph node; Red dotted arrow: short axis of lymph node.

On the second day after admission, all included patients underwent an ultrasound examination of the inguinal lymph nodes, prior to planned surgery. Preoperative ultrasonography was performed using APLIO i800 TUS-AI800 (Canon Medical Systems, USA) equipment with Ultra-Wideband Linear i18LX5 sensor by two ultrasound physicians with more than 5 years of experience. During the ultrasound, the patient was placed in a supine position and outward rotation and extension of the limbs were checked. Sufficient pressure was applied with the probe and the frequency was changed according to the patient’s body habits. Normal scanning of the vascular axis was performed first, and at least a second longitudinal scan was performed in all cases, in order to measure the two main orthogonal planes of the lymph nodes(Solivetti et al., 2012; Jacobson et al., 2015). The long and short axes of the most suspected reactive lymph nodes were measured using sonographer and the long axis was taken as a parameter to assess lymph node size (**Figure 2**).

### Statistical analysis

Statistical analysis of the data was performed using GraphPad Prism 9.0 (GraphPad Software, San Diego, California, USA). Chi-square test was used for categorical variables and Mann-Whitney analysis was used for continuous variables. Youden J statistic was used to determine the optimal threshold of inguinal lymph node size between PJI and aseptic loosening (J = sensitivity 1 specificity 1). The sensitivity and specificity of this threshold in differentiating aseptic loosening from PJI were further calculated by the following formula: Sensitivity= (true positive + true negative)/(true positive + true negative + false positive + false negative), Specificity= (true negative + false positive)/(true positive + true negative + false positive + false negative). The feasibility of inguinal lymph node size in the identification of PJI is determined by the Receiver Operator Characteristic (ROC) curves. A p-value less than 0.05 was considered statistically significant.

## Results

**Figure 3:**
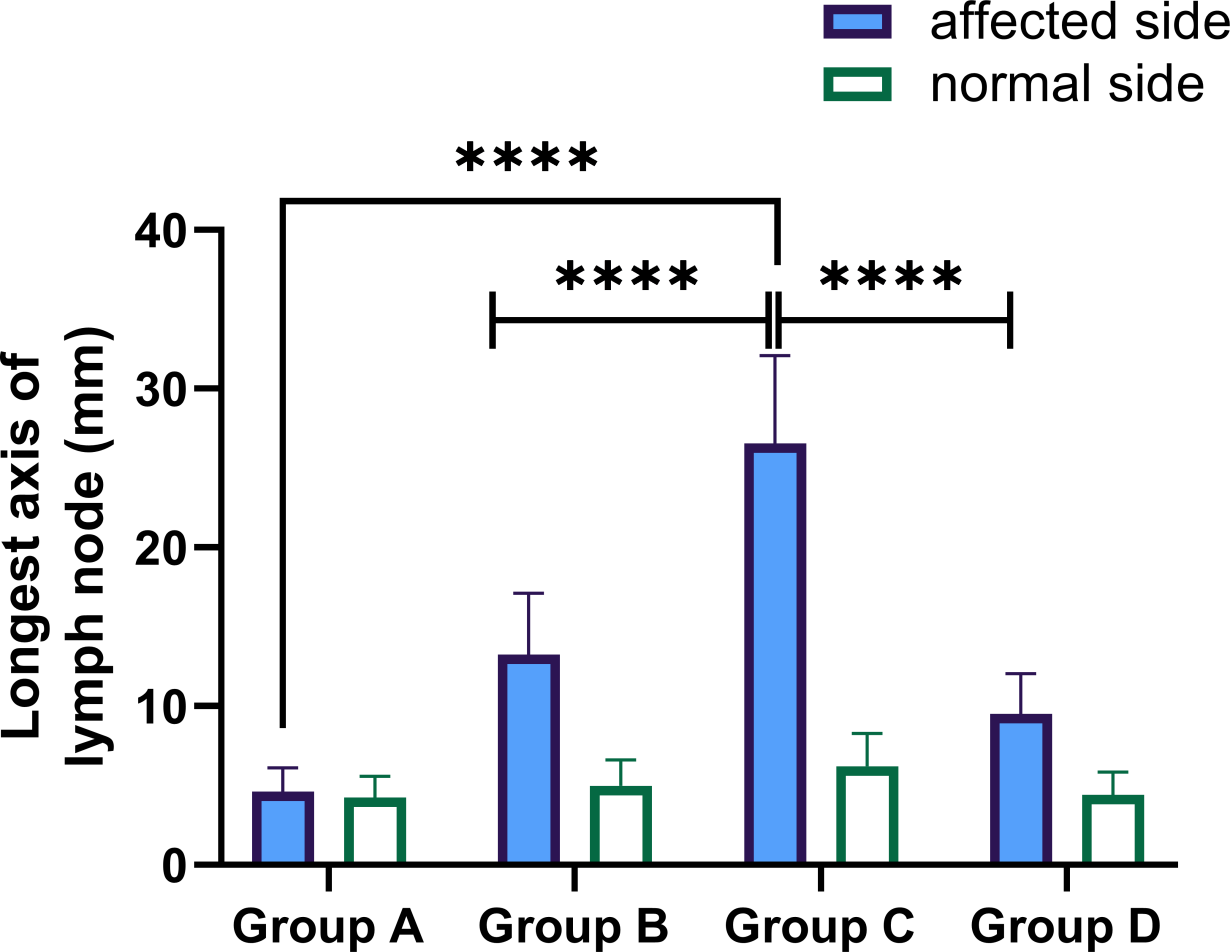
Performance of longest axis of the inguinal lymph node in the affected and normal lower limbs in each group. Group (A) = primary arthroplasty, Group (B) = aseptic revision, Group (C) = first stage of a 2-stage exchange revision protocol, Group (D) = second stage of a 2-stage exchange protocol (reimplantation), **** p-value< 0.0001.

A total of 176 patients were enrolled, including 58 patients with primary arthroplasty, 47 patients with revision for aseptic failure, 38 patients with revision for PJI and 33 patients with reimplantation. The characteristics of each group are shown in **Table 1**. No statistically significant differences were noted between the four groups concerning age, BMI, gender and involved joint. And the median time of antibiotic holiday in reimplantation group was 56 days. We then compared the size of bilateral (affected side and normal side) inguinal lymph nodes in each group and drew a histogram, as shown in **Figure 3**. It can be seen that the inguinal lymph nodes of the uninvolved lower limbs were consistent in each group, while the inguinal lymph nodes of the affected side were significantly enlarged (except for group A). The median level of inguinal lymph nodes was 26mm (range, 24 to 30mm) in the group C compared with 12 mm (range, 10 to 15mm) in the group B (p< 0.0001) (**Table 2**). The median level of inguinal lymph nodes was 4mm (range, 3.5 to 4mm) in the primary arthroplasty cohort and 9mm (range, 8 to 10mm) in the reimplantation group (**Figure 3**).

**Table 1:** Demographic data for the study population.

Characteristic	Group A (N=58)	Group B (N=47)	Group C (N=38)	Group D (N=33)	P value
Age (years)	58.5 ± 6.1	60.4 ± 5.3	61.6 ± 6.3	61.9 ± 6.4	>0.05^#^
BMI (kg/m2)	24.7 ± 2.8	24.3 ± 2.7	23.5 ± 1.9	23.9 ± 2.6	>0.05^#^
Gender					>0.05^*^
Male	27	24	17	14	
Female	31	23	21	19	
Involved Joint					>0.05^*^
Knee	32	27	21	19	
Hip	26	20	17	17	
Antibiotic Holiday (Days) median	NA	NA	NA	56	
P25, P75				(42, 84)	

Group A = primary arthroplasty, Group B = aseptic revision, Group C = first stage of a 2-stage exchange revision protocol, Group D = second stage of a 2-stage exchange protocol (reimplantation), Variables are expressed as mean ± SD (standard deviation), BMI (Body Mass Index), * Chi squared test, # Mann-Whitney U test.

**Table 2:** Comparison of parameters between each group.

Parameters	Group A	Group B	Group C	Group D	P1 value	P2 value
ESR (mm/h)						
median	11.0	21.0	35.0	12.0	0.0053	<0.0001
P25, P75	(8.0, 14.0)	(19.0, 34.0)	(18.0, 47.0)	(9.0, 16.0)		
CRP (mg/L)						
median	5	15.0	19.0	9	0.0025	<0.0001
P25, P75	(3.3, 5.9)	(5.9, 23.0)	(14.4, 25.0)	(5.1,11)		
Inguinal lymph nodes (mm)						
median	4	12.0	26.0	9	<0.001	<0.0001
P25, P75	(3.5, 4)	(10.0, 15.0)	(24.0, 30.0)	(8, 10)		

P1: group B vs group C; P2: group C vs group D; CRP, C-reactive protein; ESR, erythrocyte sedimentation rate, Group B = aseptic revision, Group C = first stage of a 2-stage exchange revision protocol.

We compared the median levels of ESR and CRP in groups B and C in **Table 2**. As can be seen, the median levels of both ESR and CRP were significantly higher in group C. The median level of ESR was 35 mm/h (range, 18 to 47 mm/h) in the group C compared with 21 mm/h (range, 19 to 34 mm/h) in the group B (p=0.0053), and the median level of CRP was 19 mg/L (range, 14.4 to 25mg/L) in group C compared with 15mg/L (range, 5.9 to 23mg/L) in group B (p=0.0025).

**Figure 4:**
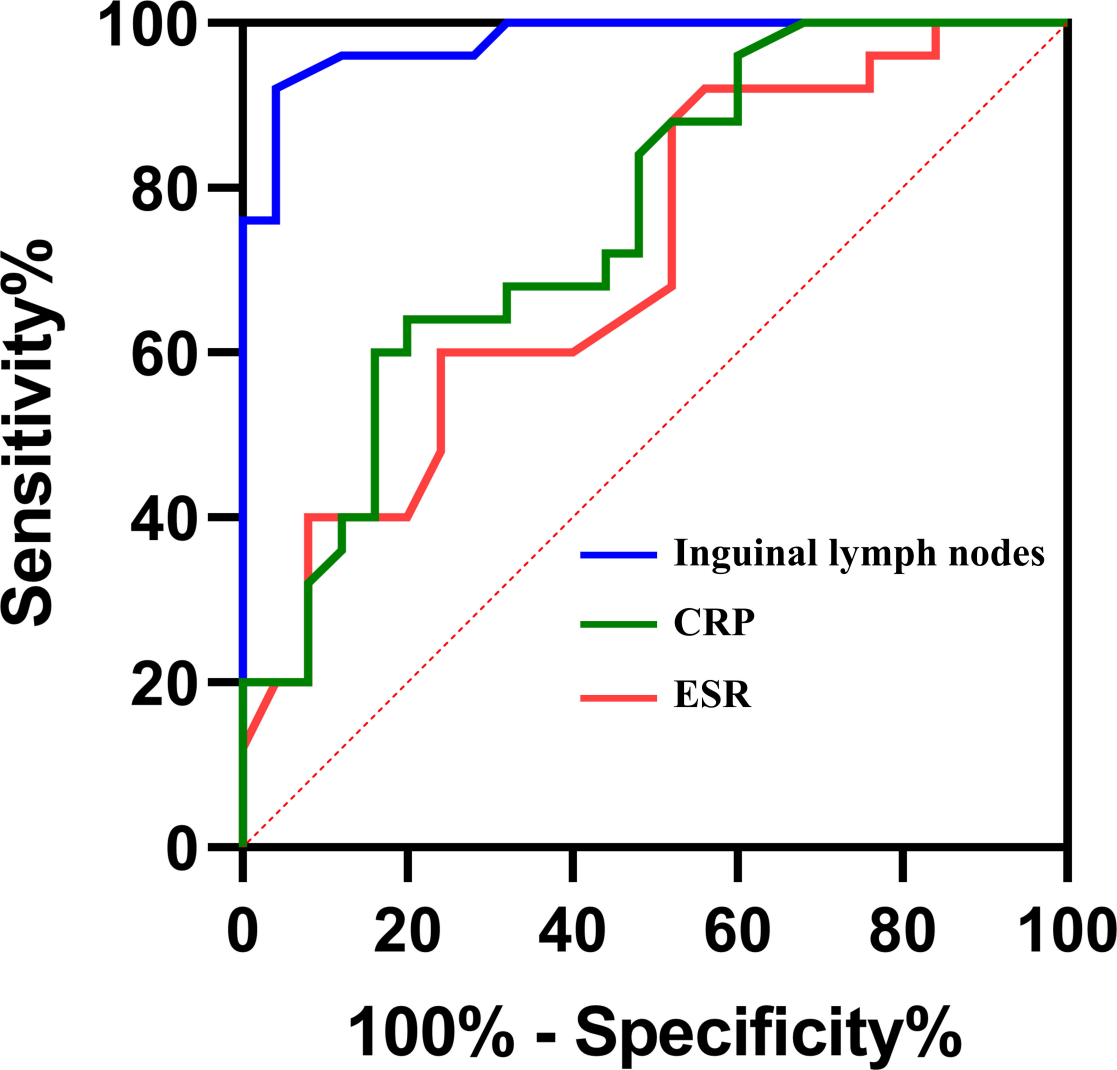
Receiver Operating Characteristic curves (ROCs) and area under curves (AUC) of the three tests in diagnosing PJI.

To further confirm the importance of inguinal lymph node size in differentiating PJI from aseptic failure, ROC curves were drawn with the PJI group as a positive reference and the aseptic loosening group as a negative control, and optimal truncation value of inguinal lymph nodes for diagnosing PJI was calculated. As shown in **Figure 4** and **Table 3**, the area under the curve (AUC) for inguinal lymph node was 0.978 (95% CI 0.946 to 1.000), and was more accurate than serum ESR 0.707 (95% CI 0.567 to 0.853) and serum CRP 0.760 (95% CI 0.628 to 0.892). The optimal threshold of inguinal lymph node size for PJI detection was 19mm, and the sensitivity, specificity and accuracy were 92%, 96% and 94%, respectively.

**Table 3:** Performance of these parameters for diagnosing PJI.

Parameters	AUC (95% CI)	Best threshold	Sensitivity (95% CI)	Specificity (95% CI)	PPV (%)	NPV (%)	Accuracy (%)
Inguinal lymph nodes	0.978 (0.946 to 1.000)	19 (mm)	92.0 (75.03 to 98.58)	96.0 (80.46 to 99.79)	95.8	92.3	94.0
CRP	0.760 (0.628 to 0.892)	18.1 (mg/L)	64.0 (44.52 to 79.75)	80.0 (60.87 to 91.14)	68.2	70.1	72.5
ESR	0.707 (0.567 to 0.853)	34.5 (mm/h)	60.0 (40.74 to 76.60)	76.0 (56.57 to 88.50)	66.7	68.0	66.0

PJI, periprosthetic joint infection, CRP, C-reactive protein; ESR, erythrocyte sedimentation rate, CI, confidence interval; PPV, positive predictive value; NPV, negative predictive value.

## Discussion

To our knowledge, this was the first study to evaluate the size of the inguinal lymph nodes by ultrasound as a diagnostic method for PJI, and also to analyze the appearance of the inguinal lymph nodes in reimplantation patients. In our cohort, inguinal lymph nodes were more accurate than ESR and CRP in diagnosing PJI. Notably, in patients with the second stage of a 2-stage exchange protocol for PJI, the size of the groin lymph nodes had largely returned to normal. This is of concern to us because there is no diagnostic gold standard for determining persistence of the infection during replantation, and often the surgeon must make a decision according to clinical symptoms and laboratory parameters (Fu et al., 2018). Besides, clinical experience has to be referred to in some cases. Previous studies have shown that serological markers such as C-reactive protein (CRP) and erythrocyte sedimentation (ESR) are unreliable markers for evaluating the persistence of reimplantation infection (George et al., 2016; Fu et al., 2018). More recently, it has been proposed that the LE strip test can be used as a reliable tool for diagnosing the persistence of infection and outperforming the serum CRP and ESR assays (Logoluso et al., 2022). However, patients in the cohort of this study were followed for less than two years after replantation which is the same as in our study, justifying the necessity of long-term. Due to the lack of long-term clinical follow-up in these patients, it is not possible to accurately determine whether the recovery of inguinal lymph node size represents the disappearance of pathogen infection. In any event, no recurrence of infection in this cohort was noticed during follow-up of fewer than 6 months.

Lymph nodes contain monocytes, macrophages, lymphatic vessels and lymph fluid, are part of the reticuloendothelial system(Mohseni et al., 2014). Lymph nodes were central to the body’s defenses against foreign antigens and function as filters, removing foreign particles from fluids that run through the lymphatic vessels(Gowing, 1974). When the pathogen is captured in the lymph nodes, it can lead to lymphocyte proliferation and enlargement, often presenting as focal lymph node enlargement in the lymphatic drainage area (Darnal et al., 2005; Mohseni et al., 2014). Inguinal lymph nodes, located in the upper part of the femoral triangle, receive lymphatic reflux material from the anterior tibial and popliteal lymph nodes, and are the first destination of lymphatic reflux of the whole lower limb (Hong et al., 2013). Localized inguinal lymphadenopathy is usually caused by infection, including STDs and pathogens in lower limb tissue (Twist and Link, 2002; Mohseni et al., 2014). In the present study, significant swelling of the inguinal lymph nodes was observed in all PJI patients. We found that when the inguinal lymph node size reached 19mm, the sensitivity and specificity of PJI were 0.92 (95%CI, 75.03 to 98.58) and 0.96 (95%CI, 80.46 to 99.79) respectively.

The current study had several strengths. First, color Doppler ultrasound has been used as a useful imaging tool to assess lymph node enlargement since the early 1970s, and ultrasound assessment of groin lymph node size is available in most medical institutions(Mountford and Atkinson, 1979; Choi et al., 1995). For patients, non-invasive and economical ultrasound testing is more acceptable. The second advantage of this study is that we prospectively compared the size of inguinal lymph nodes in patients with different disease types, reducing experimental heterogeneity. We then applied statistical methods to determine the appropriate threshold for inguinal lymph node diagnosis of PJI. While this threshold may change as data from different institutions increases, it is a good starting point and a guide for clinicians wishing to use this test. Finally, there is no doubt that the study of the timing of reimplantation in two-stage revision surgery is the most concerned topic in orthopedic infections (Shahi et al., 2017; Logoluso et al., 2022). We evaluated the appearance of inguinal lymph nodes in patients undergoing reimplantation for treatment of PJI, and there were no recurrent cases of infection in existing follow-up, although long-term follow-up results were lacking. The satisfactory results of the inguinal lymph nodes provide a possibility for decision-making on the timing of the two-stage revision, as well as a potential reference for evaluating the effectiveness of antibiotic therapy.

This study also had some limitations. First, although ultrasound is the most commonly used technique to detect inguinal lymph nodes, the operator’s operation and judgment will affect the results. The ultrasound examiners in this study were all experienced technicians with systematic training. Therefore, we believe that the results of this study are highly reliable. Second, we all know that there is no “gold standard” for diagnosing PJI, so some patients assigned to the sterile revision group may actually have PJI. Our use of the Society of Musculoskeletal Infections (MSIS) criteria to define PJI may have skewed the results, even though the MSIS criteria are generally accepted as the best definition of PJI (Parvizi and Gehrke, 2014). Finally, this study excluded patients with autoimmune inflammatory diseases such as RA, which reduced the scope of application of the findings of this study. Previous studies have shown that rheumatoid arthritis causes enlarged lymph nodes in patients (Habermann and Steensma, 2000; Mohseni et al., 2014). Therefore, inguinal lymph node examination is of reference value in patients with suspected infection of the related nodes, but in cases such as associated lower limb infection or systemic immune system disorders, the accuracy of ultrasound diagnosis may be interfered. Therefore, in future studies, we hope to explore the specific manifestations of inguinal lymph nodes in patients with inflammatory diseases.

In conclusion, the clinical application of inguinal lymph node detection adds a new powerful tool for the diagnosis of PJI. Based on our preliminary findings, we believe that ultrasound testing of inguinal lymph node size is an inexpensive, immediate, and universally available test that should be added to patient screening for PJI. In patients undergoing reimplantation, swollen lymph nodes may signal persistent pathogen infections, and of course, the interpretation of the inguinal lymph nodes in two-stage revision still requires further long-term follow-up research.

## Data availability statement

The raw data supporting the conclusions of this article will be made available by the authors, without undue reservation.

## Ethics statement

The studies involving human participants were reviewed and approved by Institutional Review Committee of the First Affiliated Hospital of Chongqing Medical University. The patients/participants provided their written informed consent to participate in this study. Written informed consent was obtained from the individual(s) for the publication of any potentially identifiable images or data included in this article.

## Author contributions

WH and NH conceived the manuscript. LQ and CZ wrote the first draft. JY and XS revised the first draft. LQ and HW performed the experiments. LC, LW, JL and CJ provided grouping suggestions and grammar modifications. All authors contributed to the article and approved the submitted version.
